# Trypanocidal Activity of Quinoxaline 1,4 Di-*N*-oxide Derivatives as Trypanothione Reductase Inhibitors

**DOI:** 10.3390/molecules22020220

**Published:** 2017-02-01

**Authors:** Karla Fabiola Chacón-Vargas, Benjamin Nogueda-Torres, Luvia E. Sánchez-Torres, Erick Suarez-Contreras, Juan Carlos Villalobos-Rocha, Yuridia Torres-Martinez, Edgar E. Lara-Ramirez, Giulia Fiorani, R. Luise Krauth-Siegel, Maria Laura Bolognesi, Antonio Monge, Gildardo Rivera

**Affiliations:** 1 Departamento de Inmunología, Escuela Nacional de Ciencias Biológicas, Instituto Politécnico Nacional, Prolongación de Carpio y Plan de Ayala, s/n, 11340 Ciudad de México, Mexico; karla.fabiola@live.com.mx (K.F.C.-V.); luviasanchez@hotmail.com (L.E.S.-T.); 2 Departamento de Parasitología, Escuela Nacional de Ciencias Biológicas, Instituto Politécnico Nacional, Prolongación de Carpio y Plan de Ayala, s/n, 11340 Ciudad de México, Mexico; bnogueda@yahoo.com (B.N.-T.); ersuco2006@yahoo.com.mx (E.S.-C.); juan_trypas@yahoo.com.mx (J.C.V.-R.); 3 Centro de Biotecnología Genómica, Instituto Politécnico Nacional, Boulevard del Maestro, s/n, Esq. Elías Piña, 88710 Reynosa, Mexico; qfbyuridia@gmail.com (Y.T.-M.); elarar0700@hotmail.com (E.E.L.-R.); 4 Center of Biochemistry, Heidelberg University, Im Neuenheimer Feld 328, 69120 Heidelberg, Germany; giulia.fiorani@studio.unibo.it (G.F.); luise.krauth-siegel@bzh.uni-heidelberg.de (R.L.K.-S.); 5 Department of Pharmacy and Biotechnology, University of Bologna, Via Belmeloro 6, 40126 Bologna, Italy; marialaura.bolognesi@unibo.it; 6 Neglected Diseases Section, Drug R&D Unit, Center for Applied Pharmacobiology Research, University of Navarra, 31008 Pamplona, Spain; amonge@unav.es

**Keywords:** isopropyl quinoxaline-7-carboxylate 1,4-di-*N*-oxide, *Trypanosoma cruzi*, trypanothione reductase inhibitors

## Abstract

Chagas disease or American trypanosomiasis is a worldwide public health problem. In this work, we evaluated 26 new propyl and isopropyl quinoxaline-7-carboxylate 1,4-di-*N*-oxide derivatives as potential trypanocidal agents. Additionally, molecular docking and enzymatic assays on trypanothione reductase (TR) were performed to provide a basis for their potential mechanism of action. Seven compounds showed better trypanocidal activity on epimastigotes than the reference drugs, and only four displayed activity on trypomastigotes; **T-085** was the lead compound with an IC_50_ = 59.9 and 73.02 µM on NINOA and INC-5 strain, respectively. An in silico analysis proposed compound **T-085** as a potential TR inhibitor with better affinity than the natural substrate. Enzymatic analysis revealed that **T-085** inhibits parasite TR non-competitively. Compound **T-085** carries a carbonyl, a CF_3_, and an isopropyl carboxylate group at 2-, 3- and 7-position, respectively. These results suggest the chemical structure of this compound as a good starting point for the design and synthesis of novel trypanocidal derivatives with higher TR inhibitory potency and lower toxicity.

## 1. Introduction

American trypanosomiasis or Chagas disease is a neglected tropical disease caused by the parasitic protozoa *Trypanosoma cruzi* [1]. The infection is transmitted to humans by blood-sucking triatomine bugs, which excrete the parasite in their feces near the bite site during feeding. Other modes of transmission are blood transfusion, congenital transmission, organ transplantation or less frequently, by eating contaminated food [2,3]. The disease is endemic in Latin America and it is estimated that around 7 million cases exist around the world [4]. Chagas disease starts with an acute phase that is frequently asymptomatic, characterized by high parasitemia that progresses to a chronic phase. In this phase, the infection may remain silent for decades and about 30% of infected individuals can develop cardiac and intestinal complications [5].

Chagas disease can be treated with nifurtimox and benznidazole [6,7]. Both are effective at the onset of the disease, but the efficacy of these drugs diminishes in the chronic phase. Currently, there is no clear consensus on the usefulness of standard therapy for treating chronic infection. Moreover, these drugs have some disadvantages; for instance, their high toxicity, their high cost, their multiple adverse effects and the need for long-term administration, which in many cases leads to abandonment of treatment. As a result of this, therapeutic failure and the emergence of resistant strains is frequent [8,9,10,11]. Therefore, the discovery of new drugs for the pharmacological treatment of Chagas disease is necessary [12]. 

Several researchers have been demonstrating the trypanocidal effect of quinoxaline derivatives. Quinoxalines are heterocyclic compounds formed by a benzene ring and a pyrazine ring. This chemical structure offers many possibilities for structural modification. It has been shown that the oxidation of both nitrogen atoms increases activity against *Mycobacterium tuberculosis* [13], *Entamoeba histolytica* [14], *Trichomonas* spp. [15], *Plasmodium falciparum* [16], *Leishmania* spp. [17] and particularly, *T. cruzi*. Cerecetto et al. [18] reported the first group of quinoxaline 1,4 di-*N*-oxide derivatives with anti-*T. cruzi* activity against the epimastigote life cycle form. They proposed that the *N*-oxide group acts as a pharmacophore. Subsequently, the trypanocidal activity of new quinoxaline *N-N*´-oxide derivatives was reported by Aguirre et al. [19], who suggested that these derivatives act as a substrate of essential enzymes of *T. cruzi,* generating oxygen reactive species harmful to the parasite. Another study by Ancizu et al. [20] reported the synthesis of carboxylic acid quinoxaline 1,4 di-*N*-oxide derivatives (CAQDO) which were further evaluated in vitro; two molecules obtained IC_50_ values similar to the reference drug nifurtimox. The study by Benitez, et al. [21] found that a halogenated substituent on the quinoxaline 1,4-di-*N*-oxide ring increased biological activity. The most recent study by Torres et al. [22] confirmed the importance of a trifluoromethyl group at R3-position, increasing trypanocidal activity and reducing host cell cytotoxicity. Our research group reported the synthesis and biological evaluation of methyl and ethyl quinoxaline-7-carboxylate 1,4-di-*N*-oxides against *T. cruzi;* three compounds (**M2**, **M6** and **M8**), showed good activity against bloodstream trypomastigotes NINOA and INC-5 of *T. cruzi* with about 50% lysis at 5 µg/mL (**M2** or **T-003** = 14.73 µM, **M8** or **T-044** = 17.98 µM and **M6** or **T-021** = 14.91 µM)*.* In the same study, molecular docking analysis suggested that these compounds could be trypanothione reductase (TR) inhibitors [23]. The contribution of methyl and ethyl ester groups in the trypanocidal activity was not clear; therefore, in this work, new propyl and isopropyl esters at 7-position on the quinoxaline ring were evaluated on epimastigotes and trypomastigotes of *T. cruzi*. Additionally, molecular docking analysis and enzymatic assays were performed on TR to confirm the mechanism of action of our derivatives.

## 2. Results and Discussion

### 2.1. Biological Activity In Vitro on Epimastigotes

In Table 1, the IC_50_, CC_50_ and SI values obtained for propyl and isopropyl quinoxaline-7-carboxylate 1,4-di-*N*-oxide derivatives against *T. cruzi* INC-5 epimastigotes are showed. Seven derivatives showed an IC_50_ less or equal to 10 µM and were more active than the reference drugs, nifurtimox and benznidazole. **T-085** was the most active compound with an IC_50_ value < 2.5 μM, while **T-069**, **T-070**, **T-071**, **T-116**, and **T-124** showed IC_50_ values ranging from 2.83 to 12.12 μM. Additionally, **T-067** was more active than benznidazole, but not nifurtimox.

A structure-activity relationship (SAR) analysis showed that the propyl derivatives had low biological activity, except compounds **T-089** and **T-124**, which possess a trifluoromethyl group at 3-position. The incorporation of this group was key in the trypanocidal activity; for example, **T-090** has a methyl group at 3-position, but when it is substituted for trifluoromethyl in **T-124**, biological activity increases 36-fold.

On the other hand, SAR analysis of isopropyl derivatives confirmed that compounds with a trifluoromethyl group at 3-position (**T-067**, **T-069**, **T-070**, **T-071**, **T-085** and **T-116**) had better biological activity than compounds that have a methyl group (**T-64**, **T-065**, **T-066**, **T-T-097**, **T-098** and **T-108**). Again, the effect of the trifluoromethyl group is evident; for example, in compounds **T-065** and **T-098**, there was a change from the methyl to trifluoromethyl group to obtain compounds **T-116** and **T-071**, respectively, enhancing considerably (>25-fold) their trypanocidal activity. These results are in agreement with a previous report by Benitez et al. [21] and Torres et al. [22] with other quinoxaline 1,4 di-*N*-oxide derivatives with trypanocidal activity. The trifluoromethyl group is stable and is related to the lipophilicity of the molecules [24] suggesting that the target of quinoxaline derivatives is internal. 

On the other hand, the compounds, **T-117** and **T-118**, have more than one trifluoromethyl group in R3 and R2, respectively; however, in excess—halogenated groups decrease epimastigote biological activity.

Also, for the isopropyl derivatives with a trifluoromethyl group at 3-position, the analysis of the effects of the substituents at 2-position was important. **T-069**, **T-071**, and **T-073** have bioisostere groups; however, **T-073**, with a furan group, showed an 8-fold decreased effect. Therefore, replacement of a sulfur atom by oxygen reduces biological activity. Additionally, the incorporation of a naphthyl group that enhances the size and polarity of the moiety **T-072**, decreases trypanocidal activity. Interestingly, compounds **T-070** and **T-085**, with a methyl and a terbutyl group at 2-position, respectively, had better trypanocidal activity.

In our previous work, the effect on trypanocidal activity between the methyl and ethyl ester group at 7-position on quinoxaline 1,4-di-*N*-oxide in analogue compounds was not conclusive [23]; however, in this study, the results showed that the change from a propyl to an isopropyl group does not confer an advantage on biological activity. The analogues compounds, **T-116** and **T-124** with a propyl and isopropyl group, respectively, showed a similar biological activity (IC_50_ 5.20 and 4.21 µM, respectively); this same biological behavior occurs between **T-069** and **T-089** (IC_50_ 4.95 and 7.59 µM, respectively). 

When analyzing the cytotoxic effect of quinoxaline derivatives on mammalian cells, the compounds **T-126** and **T-130** had the lowest cytotoxic effect; however, they were inactive on epimastigotes with both compounds having a methyl group in R3. On the other hand, **T-071** was the most toxic compound with a benzyl group in R2. 

The selectivity index (SI), which reflects the impact of the compound on the parasite, was determined by the ratio of the CC_50_ of mammalian cells and the parasite IC_50_ [25,26]. Compounds **T-070** and **T-085** showed the highest SI, becoming lead compounds from this study against epimastigotes of *T. cruzi;* both compounds have an isopropyl carboxylate group, a trifluoromethyl group, and a short aliphatic chain at R7-, R3- and R2-position, respectively. In general, all compounds showed lower selectivity than the reference drugs; therefore, we propose that rational structural modifications can be made to these molecules to decrease toxicity.

### 2.2. Biological Activity In Vitro towards Trypomastigotes T. cruzi

The compounds were evaluated in vitro against bloodstream trypomastigotes of the NINOA and INC-5 strains. Initially, all compounds were evaluated at a single concentration (50 µg/mL) to identify molecules with the best activity against this stage of the parasite and to select those that induce at least 50% lysis. Compounds with a better lysis percentage on both strains were **T-069**, **T-071**, **T-085** and **T-089**. The compound **T-085** showed a better trypanocidal activity than the reference drugs against trypomastigotes (Table 2). However, in future studies, **T-085** needs to be tested against the amastigote life cycle stage of the parasite to know its biological effects in this form.

Epimastigotes were more sensitive under the conditions tested to quinoxaline 1,4 di-*N*-oxide derivatives than bloodstream trypomastigotes; only half of the compounds that showed activity against epimastigotes were active against trypomastigotes.

Compounds **T-067**, **T-070**, **T-116** and **T-124** showed good activity on epimastigotes, but significantly reduced their activity on bloodstream trypomastigotes. **T-067**, **T-116** and **T-124** have a carboxylate group at R2- and compound **T-070** has a carbonyl group at R2-, followed by a primary carbon. Interestingly, the four compounds that were active against bloodstream trypomastigotes have a carbonyl group followed by a tertiary carbon instead. The previous SAR analysis highlights the importance of maintaining a carbonyl group in R2 to conserve activity in both stages of the parasite.

In the life cycle of *T. cruzi*, the epimastigote form is found in the vector and the bloodstream trypomastigote form in the mammal [1], each biological form of the parasite requires different culture conditions, therefore the methodology used to evaluate the effect of the compounds and the results are not the same. Evaluation in bloodstream trypomastigotes is important because it is the infectious form in mammals and is the main form in acute phase of the infection where parasitemia is high. As mentioned, experimental assays in both parasites forms, display different information. In this work, the ability of the compounds to lyse trypomastigotes was measured while the viability of epimastigotes following compounds exposure was determined. In trypomastigotes, the effect on cell proliferation cannot be determined because in this phase the parasite does not divide; however, the effect on cell lysis, loss of mobility or loss of infectivity can be evaluated [25]. 

### 2.3. In Silico Binding Prediction

**Figure 1:**
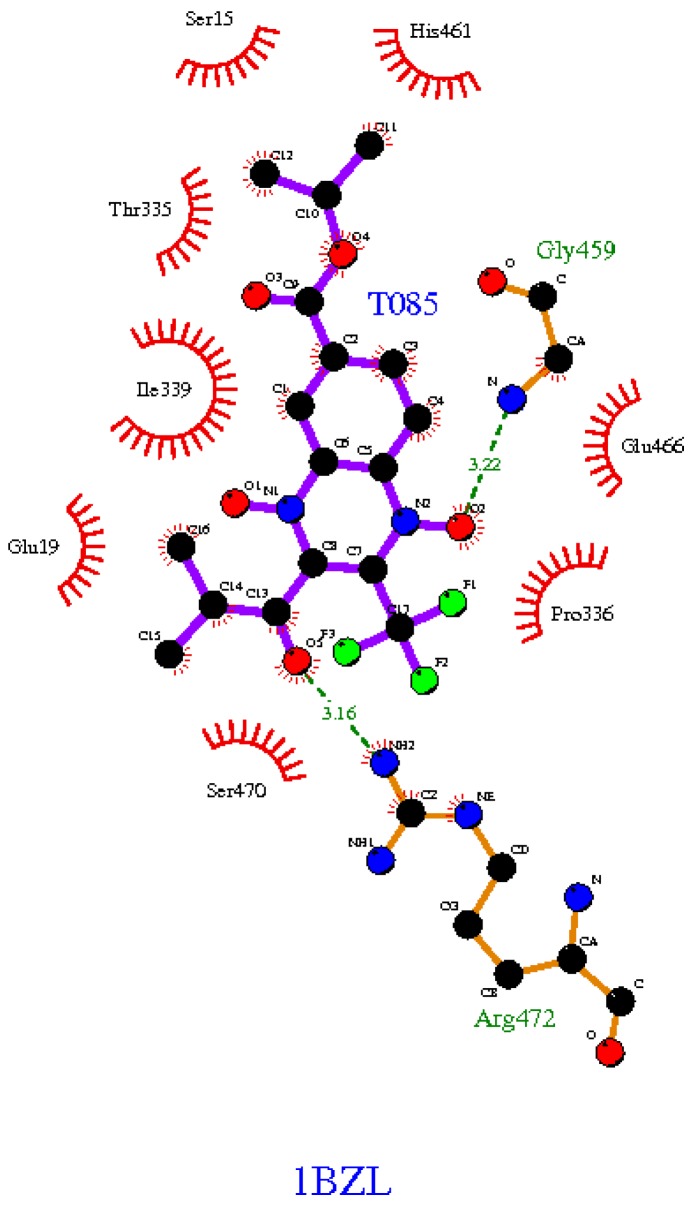
2D visualization of the compound **T-085** docked on the binding pocket of TR (PDB ID: 1BZL). Red lines represent hydrophobic interaction of non-ligand residues, green dashed lines represents hydrogen amino acid-ligand interaction and its length, black balls joined with blue balls represent ligand bonds, green balls represents the 3F atoms. The plot was created with LigPlot + software [28].

The prediction values of the binding-energy of quinoxaline 1,4-di-*N*-oxide derivatives docked in the TR protein are shown in Table 3. In this analysis, we added three compounds that our research group previously reported as potential TR inhibitors (**T-003** or **M2**, **T-021** or **M6** and **T-044** or **M8**) [23]. A total of 16 compounds showed a higher binding-energy than the natural substrate trypanothionine (range: from −5.9 to −6.7 Kcal/mol) and 13 compounds showed the lowest binding energy (range: from −6.9 to −8.6 Kcal/mol). The compounds with the highest free energy (in comparison with trypanothionine) also had the lowest molecular weights (mean = 337.01, S.D. 42.47), and the compounds with the best binding affinity had the highest molecular weights (mean = 403.44, S.D. 48.24). These were statistically significant in an independent-sample t test (t = −4.1091, df = 28.168, *p*-value = 0.0003). This statistical analysis indicates that compounds with better binding energy are influenced by a summation of atom-pair interaction (Vina score calculation) caused by their high MW. In addition, a Spearman correlation analysis (r = −0.08, *p* = 0.77) was not significant, showing that the predicted affinity for those compounds is due to specificity and not to the size of the molecule [27]. On the other hand, in these results, it is evident that the predicted binding energy follows a similar behavior to the in vitro analysis. It is interesting that among the compounds with the best binding energy, there are some isopropyl derivatives with a trifluoromethyl group at 3-position that in SAR analysis have the best biological activity. For example, compound **T-085** showed notable in vitro trypanocidal effects in epimastigotes and trypomastigotes. It has been reported that the essential residues involved in the catalysis of the TR protein are Cys53 and Cys58 and the active-site base His461 [27]. Thus, we inspect the interactions of the best compound **T-085** with a focus on those essential amino acids (Figure 1). **T-085** is in contact thorough hydrogen bonds with two amino acids, and through hydrophobic bonds with eight amino acids; among these, the essential catalytic His461. Thus, this compound is probably interfering with the TR-trypanothione disulfide binding process, but this needs further enzymatic validation.

### 2.4. TR Inhibition

**Figure 2:**
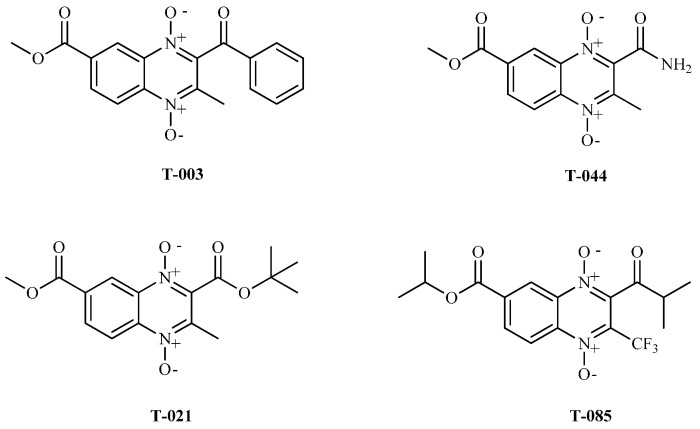
Structure of quinoxaline-7-carboxylate derivatives tested as TR inhibitors. **T-003**, **T-021** and **T-044** were previously reported with trypanocidal activity by Villalobos-Rocha et al.

Based on previous reports [23] and previous docking analysis, we suggested that quinoxaline-7-carboxylate-1,4-di-*N*-oxide derivatives could bind and inhibit TR. This molecular mechanism of action might explain the observed antiparasitic activity against *T. cruzi*. On this basis, three methyl (**T-003**, **T-021**, and **T-044**) and one isopropyl (**T-085**) quinoxaline-7-carboxylate 1,4-di-*N*-oxide derivatives (Figure 2) were selected to test their ability to inhibit TR, using a biochemical assay [29]. 

In the first step, the compounds were studied at a fixed concentration of 100 μM or 40 μM of substrate TS_2_. Under these conditions, only the quinoxaline **T-085** proved to be a TR inhibitor. At 60 µM, the highest concentration that could be measured in the assay, **T-085,** showed 60% inhibition (Table 4). **T-021** and **T-044** displayed no or negligible inhibitory activity, even when tested at 100 μM; however, these compounds had trypanocidal activity previously [23]. This suggests another mechanism of action for their biological effect.

**Figure 3:**
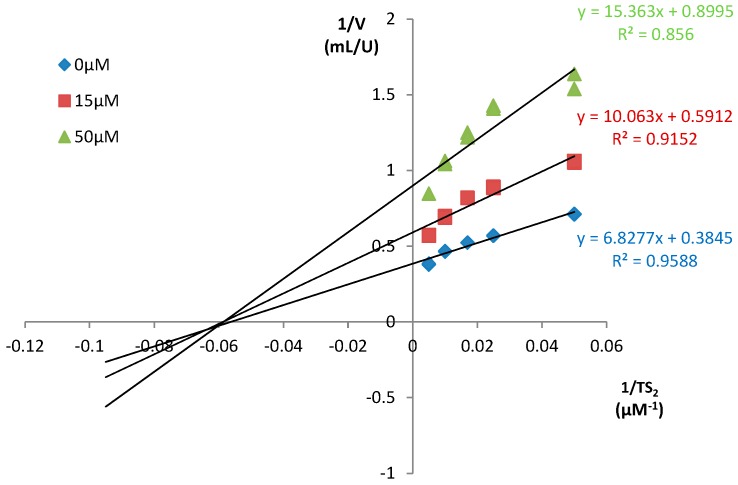
Lineweaver–Burk plot for the inhibition of *Tc*TR by **T-085**. The activity of the enzyme was measured in the absence and presence of two fixed concentrations of inhibitor (15 µM and 50 µM) and varying the concentration of the substrate (200 µM, 100 µM, 60 µM, 40 µM and 20 µM). The inhibitory constant Ki was calculated from the direct plot using nonlinear least-squares data fitting in an Excel spreadsheet.

From these results, we concluded that the quinoxaline-7-carboxylate-1,4-di-*N*-oxide scaffold can be a suitable motif for TR recognition only when a proper group is attached at 2-position. In fact, the carboxamide **T-044** and the tert-butyl ester **T-021** did not inhibit the enzyme, whereas the ketone **T-085** displayed a significant inhibition. Furthermore, the percentages of inhibition were virtually identical at the two substrate concentrations (Table 4). This suggested an inhibitory mechanism independent of the substrate concentration. Indeed, a Lineweaver-Burk plot revealed **T-085** as a non-competitive inhibitor with a Ki of 35.0 μM (Figure 3). The Ki-value for these compounds is comparable to those of other known TR inhibitors such as mepacrine (Ki = 19 μM).

**Figure 4:**
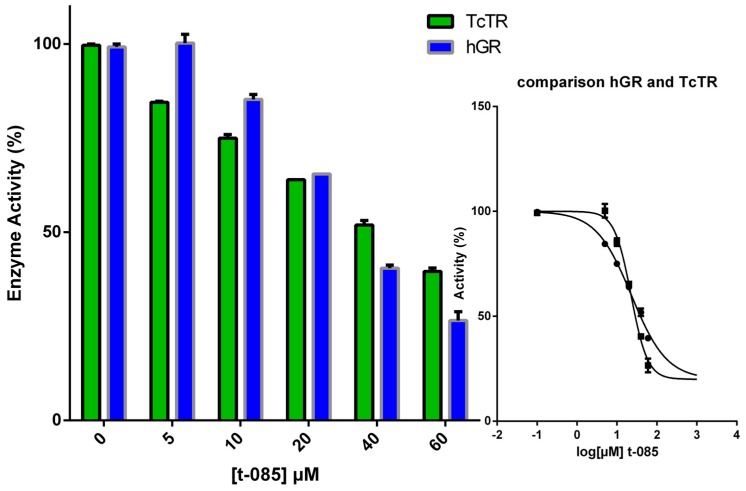
Comparison of the *Tc*TR (green bars and circles) and of hGR (blue bars and squares) inhibition by **T-085** (0–60 µM). The activity of both enzymes was measured by following NADPH consumption at 340 nm as described in the experimental section. The enzyme activity refers to that of the enzyme in the presence of DMSO. Data are means ± SD of triplicate determinations.

To assess selectivity over the human homologue glutathione reductase (GR), **T-085** was studied at a fixed concentration (5, 10, 20, 40 and 60 μM) against both enzymes and the results are graphically depicted in Figure 4. Regrettably, compound **T-085** has a similar IC_50_ value of 50 µM against TcTR and hGR. The collected results suggest that **T-085** is a good starting point for future medicinal chemistry studies to identify novel derivatives with higher TR inhibitory potency, an improved selectivity profile over the host GR and lower toxicity.

## 3. Materials and Methods

### 3.1. Synthetic Compounds

Propyl and isopropyl quinoxaline-7-carboxylate 1,4-di-*N*-oxide derivatives were synthetized as described by Gomez-Caro et al. [30]. The compounds were characterized by infrared (IR), nuclear magnetic resonance (NMR), and elemental analysis. Stock solutions of the compounds were prepared in dimethyl sulfoxide (DMSO; Sigma-Aldrich, Toluca, Edo de Mexico, Mexico), the subsequent serial dilutions were made in phosphate-buffered saline (PBS). The final DMSO concentration did not exceeded 1% [31]. Nifurtimox (Nfx: Lampit™, Bayer, Lot: 12060199, El Salvador) and benznidazol (Bnz; Rochagan™, Roche, Lot: 110878, Rio de Janeiro, Brazil) were used as reference drugs.

#### Chemical Compounds

**T-064**: *Isopropyl methyl-3-methyl-quinoxaline-2,7-dicarboxylate 1,4-di-N-oxide*. This compound was obtained in 7.9% yield from isopropyl benzofuroxane-5-carboxylate and methyl acetoacetate. IR (KBr): 2986 (C-H), 1744 and 1712 (C=O), 1330 (*N*-oxide) cm^−1^. ^1^H-NMR (400 MHz, DMSO-*d*_6_) δ ppm: 1.38 (s, 6H, (CH_3_)_2_CH), 2.43 (s, 3H, CH_3_), 4.03 (s, 3H, COOCH_3_), 5.21–5.25 (m, (CH_3_)_2_-CH-), 8.34 (d, *J* = 7.04 Hz, 1H, H5), 8.51 (d, *J* = 6.60 Hz, 1H, H6), 8.88 (s, 1H, H8). Calculated analysis for C_15_H_16_N_2_O_6_: C, 56.25; H, 5.04; N, 8.75. Found: C, 56.10; H, 4.75; N, 8.35

**T-065**: *Isopropyl ethyl-3-methylquinoxaline-2,7-dicarboxylate 1,4-di-N-oxide*. This compound was obtained in 3.5% yield from isopropyl benzofuroxane-5-carboxylate and ethyl acetoacetate. IR (KBr): 2984 (C-H), 1740 and 1710 (C=O), 1332 (*N*-oxide) cm^−1^. ^1^H-NMR (400 MHz, DMSO-*d*_6_) δ ppm: 1.39 (s, 9H, (CH_3_)_2_CH and CH_2_CH_3_), 2.44 (s, 3H, CH_3_), 4.51 (d, *J* = 6.30 Hz, 2H, CH_2_CH_3_), 5.22–5.25 (m, (CH_3_)_2_-CH-), 8.34 (d, *J* = 8.75 Hz, 1H, H5), 8.51 (d, *J* = 8.37 Hz, 1H, H6), 8.85 (s, 1H, H8). Calculated analysis for C_16_H_18_N_2_O_6_: C, 57.48; H, 5.43; N, 8.38. Found: C, 57.10; H, 5.05; N, 7.90.

**T-066**: *Isopropyl tert-butyl-3-methylquinoxaline-2,7-dicarboxylate 1,4-di-N-oxide*. This compound was obtained in 20% yield from isopropyl benzofuroxane-5-carboxylate and *tert*-butyl acetoacetate. IR (KBr): 2978 (C-H), 1736 and 1711 (C=O), 1331 (*N*-oxide) cm^−1^. ^1^H-NMR (400 MHz, DMSO-*d*_6_) δ ppm: 1.38 (s, 6H, (CH_3_)_2_CH), 1.61 (s, 9H, COOC(CH_3_)_3_), 2.46 (s, 3H, CH_3_), 5.22–5.25 (m, 1H, (CH_3_)_2_CH), 8.37 (d, *J* = 9.02 Hz, 1H, H5), 8.54 (m, 1H, H6), 8.86 (s, 1H, H8). Calculated analysis for C_18_H_22_N_2_O_6_: C, 59.66; H, 6.12; N, 7.73. Found: C, 59.42; H, 5.82; N, 7.56.

**T-067**: *Isopropyl ethyl-3-(2-ethoxy-2-oxoethyl) quinoxaline-2,7-dicarboxylate 1,4-di-N-oxide*. This compound was obtained in 5.4% yield from isopropyl benzofuroxane-5-carboxylate and diethyl 3-oxoglutarate. IR (KBr): 2988 (C-H), 1740 and 1719 (C=O), 1328 (*N*-oxide) cm^−1^. ^1^H-NMR (400 MHz, DMSO-*d*_6_) δ ppm: 1.15 (s, 3H, CH_2_COOCH_2_CH_3_), 1.30 (s, 3H, COOCH_2_CH_3_), 1.36 (m, 6H, (CH_3_)_2_CH-), 3.77 (s, 2H, CH_2_COOCH_2_CH_3_), 4.10 (d, *J*_1_ = 7.21 Hz, 2H, COOCH_2_CH_3_), 4.48 (d, *J*_1_ = 6.76 Hz, 2H, CH_2_COOCH_2_CH_3_), 5.19–5.22 (m, 1H, (CH_3_)_2_CH-), 8.40 (d, *J* = 9.13 Hz, 1H, H5), 8.53 (d, *J* = 9.42 Hz, 1H, H6), 8.85 (s, 1H, H8). Calculated analysis for C_19_H_22_N_2_O_8_: C, 56.15; H, 5.46; N, 6.89. Found: C, 56.20; H, 5.20; N, 6.40.

**T-069**: *Isopropyl 2-(thiophene-2-carbonyl)-3-trifluoromethylquinoxaline-7-carboxylate 1,4-di-N-oxide*. This compound was obtained in 2.5% yield from isopropyl benzofuroxane-5-carboxylate and 4,4,4-trifluoro-1-(2-thienyl)-1,3-butanedione. IR (KBr): 2968 (C-H), 1721 and 1661 (C=O), 1332 (*N*-oxide), 1285 and 1153 (Ar-CF_3_) cm^−1^. ^1^H-NMR (400 MHz, DMSO-*d*_6_) δ ppm: 1.40 (m, 6H, (CH_3_)_2_CH), 5.24–5.29 (m, (CH_3_)_2_-CH-), 7.31 (s, 1H, H4, C_4_H_3_S), 8.24 (s, H5, C_4_H_3_S), 8.30 (s, H3, C_4_H_3_S), 8.51 (s, 1H, H5), 8.54 (s, Hz, 1H, H6), 8.94 (s, 1H, H8). Calculated analysis for C_18_H_13_F_3_N_2_O_5_S: C, 50.71; H, 3.07; N, 6.57. Found: C, 50.30; H, 2.85; N, 6.21.

**T-070**: *Isopropyl 2-acetyl-3-trifluoromethylquinoxaline-7-carboxylate 1,4-di-N-oxide*. This compound was obtained in 8.0% yield from isopropyl benzofuroxane-5-carboxylate and 1,1,1-trifluoro-2,4-pentanedione. IR (KBr): 2988 (C-H), 1726 (C=O), 1337 (*N*-oxide), 1285 and 1153 (Ar-CF_3_) cm^−1^. ^1^H-NMR (400 MHz, DMSO-*d*_6_) δ ppm: 1.41 (s, 6H, (CH_3_)_2_CH), 2.62 (s, 3H, COCH_3_), 5.22–5.28 (m, (CH_3_)_2_-CH-), 8.5 (d, *J* = 10.34 Hz, 1H, H5), 8.6 (d, *J* = 8.94 Hz, 1H, H6), 8.9 (s, 1H, H8). Calculated analysis for C_15_H_13_F_3_N_2_O_5_: C, 50.29; H, 3.66; N, 7.82. Found: C, 49.88; H, 3.30; N, 7.56.

**T-071**: *Isopropyl 2-benzoyl-3-trifluoromethylquinoxaline-7-carboxylate 1,4-di-N-oxide*. This compound was obtained in 7.5% yield from isopropyl benzofuroxane-5-carboxylate and 4,4,4-trifluoro-1-phenyl-1,3-butanedione. IR (KBr): 2997 (C-H), 1720 and 1683 (C=O), 1328 (*N*-oxide), 1292.49 and 1162 (Ar-CF_3_) cm^−1^. ^1^H-NMR (400 MHz, DMSO-*d*_6_) δ ppm: 1.42 (s, 6H, (CH_3_)_2_CH), 5.24–5.29 (m, (CH_3_)_2_-CH-), 7.6 (d, *J* = 7.16 Hz, 2H, H3 and H5, C_6_H_5_), 7.77 (s, 1H, H4, C_6_H_5_), 8.16 (d, *J* = 7.17 Hz, 2H, H2 and H6, C_6_H_5_), 8.51–8.53 (m, 2H, H5 and H6), 8.95 (s, 1H, H8). Calculated analysis for C_20_H_15_F_3_N_2_O_5_: C, 57.15; H, 3.60; N, 6.66. Found: C, 56.90; H, 3.18; N, 6.21.

**T-072**: *Isopropyl 2-(naphthyl-2-carbonyl)-3-trifluoromethylquinoxaline-7-carboxylate 1,4-di-N-oxide*. This compound was obtained in 4.5% yield from isopropyl benzofuroxane-5-carboxylate and 4,4,4-trifluoromethyl-1-(2-naphthyl)-1,3-butanedione. IR (KBr): 2981 (C-H), 1724 and 1673 (C=O), 1336 (*N*-óxido), 1282 and 1172 (Ar-CF_3_) cm^−1^. ^1^H-NMR (400 MHz, DMSO-*d*_6_) δ ppm: 1.42 (s, 6H, (CH_3_)_2_CH), 5.25-5.29 (m, (CH_3_)_2_-CH-), 7.66 (t, *J* = 7.15 Hz, 1H, H3, C_10_H_7_), 7.75 (t, *J* = 7.19 Hz, 1H, H6, C_10_H_7_), 8.0 (d, *J* = 8.08 Hz, 1H, H7, C_10_H_7_), 8.1 (d, *J* = 87.99 Hz, 1H, H5, C_10_H_7_), 8.14 (s, 2H, H2, and H4 C_10_H_7_), 8.5 (d, *J* = 7.59 Hz, 2H, H5 and H6), 8.87 (s, 1H, H8), 9.0 (s, 1H, H8, C_10_H_7_). Calculated analysis for C_24_H_17_F_3_N_2_O_5_: C, 61.28; H, 3.64; N, 5.96. Found: C, 61.01; H, 3.35; N, 5.62.

**T-073**: *Isopropyl 2-(furyl-2-carbonyl)-3-trifluoromethylquinoxaline-7-carboxylate 1,4-di-N-oxide*. This compound was obtained in 17.30% yield from isopropyl benzofuroxane-5-carboxylate and 4,4,4-trifluoro-1-(2-furyl)-1,3-butanedione. IR (KBr): 2993 (C-H), 1714 and 1664 (C=O), 1332 (*N*-oxide), 1255 and 1168 (Ar-CHF_2_) cm^−1^. ^1^H-NMR (400 MHz, DMSO-*d*_6_) δ ppm: 1.40 (s, 6H, (CH_3_)_2_CH), 5.23–5.29 (q, *J*_1_ = 6.27 Hz, *J*_2_ = 12.45 Hz, (CH_3_)_2_-CH-), 6.84 (s, 1H, H4, C_4_H_3_O), 7.9 (d, *J* = 3.73 Hz, 1H, H5, C_4_H_3_O), 8.26 (s, 1H, H3, C_4_H_3_O), 8.5 (d, *J* = 8.97 Hz, 1H, H5), 8.6 (d, *J* = 8.89 Hz, 1H, H6), 8.94 (s, 1H, H8). Calculated analysis for C_18_H_13_F_3_N_2_O_6_: C, 52.69; H, 3.19; N, 6.83. Found: C, 52.36; H, 2.74; N, 6.41.

**T-085**: *Isopropyl 2-isobutyryl-3-trifluoromethylquinoxaline-7-carboxylate 1,4-di-N-oxide*. This compound was obtained in 16.0% yield from isopropyl benzofuroxane-5-carboxylate and 1,1,1-trifluoromethyl-5-methyl-2,4-hexanedione. IR (KBr): 2983 (C-H), 1715 and 1678 (C=O), 1325 (*N*-oxide), 1283 and 1178 (Ar-CF_3_) cm^−1^. ^1^H-NMR (400 MHz, DMSO-*d*_6_) δ ppm: 1.30 (s, 6H, COCH(CH_3_)_2_), 1.33 (s, 6H, (CH_3_)_2_CH), 1.4 (d, *J*_1_ = 6.25 Hz, 1H, COCH(CH_3_)_2_), 5.25 (q, *J*_1_ = 6.41 Hz, *J*_2_ = 12.54 Hz, (CH_3_)_2_-CH-), 8.5 (d, *J* = 8.94 Hz, 1H, H5), 8.6 (d, *J* = 8.88 Hz, 1H, H6), 8.88 (s, 1H, H8). Calculated analysis for C_17_H_17_F_3_N_2_O_5_: C, 52.85; H, 4.44; N, 7.25. Found: C, 52.40; H, 4.12; N, 7.04.

**T-088**: *n-Propil methyl-3-methylquinoxaline-2,7-dicarboxylate 1,4-di-N-oxide*. This compound was obtained in 11.85% yield from *n*-propil benzofuroxane-5-carboxylate and methyl acetoacetate. IR (KBr): 2964.93 (ArC-H), 1745.58 (C=O), 1331 (*N*-oxide) cm^−1^. ^1^H-NMR (400 MHz, DMSO-*d*_6_) δ ppm: 1.0 (t, 3H, CH_3_(CH_2_)_2_O), 1.74–1.84 (m, 2H, CH_3_(CH_2_)_2_O), 2.44 (3H, CH_3_), 4.03 (s, 3H, COOCH_3_), 4.32–4.36 (m, 2H, CH_3_(CH_2_)_2_O), 8.35–8.40 (m, 1H, H5), 8.51–8.57 (m, 1H, H6), 8.85–8.90 (m, 1H, H8). Calculated analysis for C_15_H_16_N_2_O_6_: C, 56.25; H, 5.04; N, 8.75. Found: C, 56.1; H, 4.9; N, 8.4.

**T-089**: *n-Propil 2-(thiophene-2-carbonyl)-3-trifluoromethylquinoxaline-7-carboxylate 1,4-di-N-oxide*. This compound was obtained in 6.51% yield from *n*-propil benzofuroxane-5-carboxylate and 4,4,4-trifluoro-1-(2-thienyl)-1,3-butanedione. IR (KBr): 2965 (ArC-H), 1722 and 1662 (C=O), 1331 (*N*-oxide), 1284 and 1165 (Ar-CF_3_) cm^−1^. ^1^H-NMR (400 MHz, DMSO-*d*_6_) δ ppm: 1.0 (t, 3H, CH_3_(CH_2_)_2_O), 1.77–1.86 (m, 2H, CH_3_(CH_2_)_2_O), 4.37–4.40 (m, 3H, CH_3_(CH_2_)_2_O), 7.30–7.34 (m, 1H, C_4_H_3_S), 8.25 (m, 1H, C_4_H_3_S), 8.31 (m, 1H, C_4_H_3_S), 8.5–8.7 (m, 2H, H5, H6), 8.96 (s, 1H, H8). Calculated analysis for C_18_H_13_F_3_N_2_O_5_S: C, 50.71; H, 3.07; N, 6.57. Found: C, 50.35; H, 2.85; N, 6.26.

**T-090**: *n-Propil ethyl-3-methylquinoxaline-2,7-dicarboxylate 1,4-di-N-oxide*. This compound was obtained in 17.85% yield from n-propil benzofuroxane-5-carboxylate and ethyl acetoacetate. IR (KBr): 2965 (ArC-H), 1742.53 and 1721.70 (C=O), 1329 (*N*-oxide) cm^−1^. ^1^H-NMR (400 MHz, DMSO-*d*_6_) δ ppm: 1.01 (t, 3H, CH_3_(CH_2_)_2_O), 1.36 (t, 3H, COOCH_2_CH_3_), 1.76–1.82 (q, *J* = 7.11, *J* = 14.15, 2H, CH_3_(CH_2_)_2_O), 2.45 (s, 3H, CH_3_), 4.32–4.36 (m, 2H, CH_3_(CH_2_)_2_O), 4.49–4.54 (q, *J* = 7.09 2H, COOCH_2_CH_3_), 8.35–8.40 (m, 1H, H5), 8.5 (q, *J* = 9.0 Hz, *J* = 16.06 Hz, 1H, H6), 8.9 (s, 1H, H8). Calculated analysis for C_16_H_18_N_2_O_6_: C, 57.48; H, 5.43; N, 8.38. Found: C, 57.20; H, 5.18; N, 8.15.

**T-091**: *n-Propil tert-butyl-3-methylquinoxaline-2,7-dicarboxylate 1,4-di-N-oxide*. This compound was obtained in 7.58% yield from *n*-propil benzofuroxane-5-carboxylate and tert-butyl acetoacetate. IR (KBr): 2966 (ArC-H), 1740 and 1721 (C=O), 1325 (*N*-oxide) cm^−1^. ^1^H-NMR (400 MHz, DMSO-*d*_6_) δ ppm: 1.01 (t, 3H, CH_3_(CH_2_)_2_O), 1.61 (s, 9H, COOC(CH_3_)_3_), 1.8 (q, *J*_1_ = 6.98, *J*_2_ = 13.98 Hz, 2H, CH_3_(CH_2_)_2_O), 2.46 (s, 3H, CH_3_), 4.33–4.36 (m, 3H, 2H, CH_3_(CH_2_)_2_O), 8.4 (d, *J* = 9.47 Hz, 1H, H5), 8.6 (q, *J* = 8.93, *J* = 16.26 Hz, 1H, H6), 8.92 (s, 1H, H8). Calculated analysis for C_18_H_22_N_2_O_6_: C, 59.66; H, 6.12; N, 7.73. Found: C, 59.30; H, 5.80; N, 7.45.

**T-097**: *Isopropyl 2-phenylamide-3-methylquinoxaline-7-carboxylate 1,4-di-N-oxide*. This compound was obtained in 18.5% yield from isopropyl benzofuroxane-5-carboxylate and 3-oxo-*N*-phenylbutanamide. IR (KBr): 2984 (C-H), 1720 and 1680 (C=O), 1332 (*N*-oxide) cm^−1^. ^1^H-NMR (400 MHz, DMSO-*d*_6_) δ ppm: 1.39 (s, 6H, (CH_3_)_2_CH), 2.90 (s, 1H, CH_3_), 5.23 (q, *J*_1_ = 6.41 Hz, *J*_2_ = 12.68 Hz, (CH_3_)_2_-CH-), 7.20 (t, *J* = 7.38 Hz, 1H, H4-NHC_6_H_5_), 7.4 (t, *J* = 7.82 Hz, 2H, H3 and H5, NHC_6_H_5_), 7.8 (d, *J* = 7.9 Hz, H2 and H6, NHC_6_H_5_), 8.17–8.30 (m, 2H, H5 and H6), 8.73 (s, 1H, H8), 10.85 (s, 1H, NH). Calculated analysis for C_20_H_19_N_3_O_5_: C, 62.99; H, 5.02; N, 11.02. Found: C. 62.56; H, 4.87; N, 10.70.

**T-098**: *Isopropyl 2-benzoyl-3-methylquinoxaline-7-carboxylate 1,4-di-N-oxide*. This compound was obtained in 45% yield from isopropyl benzofuroxane-5-carboxylate and 1-phenyl-1,3-butanedione. IR (KBr): 2983 (C-H), 1715 and 1678 (C=O), 1326 (*N*-oxide) cm^−1^. ^1^H-NMR (400 MHz, DMSO-*d*_6_) δ ppm: 1.40 (s, 6H, (CH_3_)_2_CH), 2.30 (s, 3H, CH_3_), 5.3 (q, *J*_1_ = 6.23 Hz, *J*_2_ = 12.30 Hz, (CH_3_)_2_-CH-), 7.6 (t, *J* = 7.84 Hz, 2H, H3 and H5, C_6_H_5_), 7.8 (t, *J* = 7.95 Hz, 1H, H4, C_6_H_5_), 8.1 (d, *J* = 8.28 Hz, 2H, H2 and H6, C_6_H_5_), 8.4 (d, *J* = 8.4 Hz, 1H, H5), 8.5 (d, *J* = 8.95 Hz, 1H, H6), 8.97 (s, 1H, H8). Calculated analysis for C_20_H_18_N_2_O_5_: C, 65.57; H, 4.95; N, 7.65. Found: C, 65.23; H, 4.47; N, 7.32.

**T-107**: *Isopropyl 2-(tert-butyl-2-carbonyl)-3-tert-butylquinoxaline-7-carboxylate 1,4-di-N-oxide*. This compound was obtained in 5% yield from isopropyl benzofuroxane-5-carboxylate and 2,2,6,6-tetramethyl-3,5-heptanedione. IR (KBr): 2978 (C-H), 1736 and 1711 (C=O), 1331 (*N*-oxide) cm^−1^. ^1^H NMR (400 MHz, DMSO-*d*_6_) δ ppm: 1.34 (s, 9H, C(CH_3_)_3_), 1.35 (s, 9H, COC(CH_3_)_3_), 1.38 (s, 6H, (CH_3_)_2_CH), 5.13–519 (m, (CH_3_)_2_-CH-), 7.96 (d, *J* = 9.52 Hz, 1H, H5), 8.17 (d, *J* = 9.62 Hz, 1H, H6), 8.67 (s, 1H, H8). Calculated analysis for C_21_H_28_N_2_O_5_: C, 64.93; H, 7.27; N, 7.21. Found: C, 64.73; H, 7.03; N, 6.95.

**T-108**: *Isopropyl 2-amide-3-methyl-quinoxaline-7-carboxilate 1,4-di-N-óxide*. This compound was obtained in 4.0% yield from isopropyl benzofuroxane-5-carboxylate and methyl acetoacetamide. IR (KBr): 2982 (C-H), 1738 and 1714 (C=O), 1328 (*N*-oxide) cm^−1^. ^1^H-NMR (400 MHz, DMSO-*d*_6_) δ ppm: 1.38 (s, 6H, (CH_3_)_2_CH), 2.48 (s, 3H, CH_3_), 5.20-2.27 (m, (CH_3_)_2_-CH-), 8.20 (s, 2H, CONH_2_), 8.38 (d, *J* = 9.17 Hz, 1H, H5), 8.58 (d, *J* = 8.77 Hz, 1H, H6), 8.90 (s, 1H, H8). Calculated analysis for C_14_H_16_N_3_O_5_: C, 55.08; H, 4.95; N, 13.76. Found: C, 54.76; H, 4.36; N, 13.35

**T-116**: *Isopropyl ethyl-3-trifluoromethylquinoxaline-2,7-dicarboxylate 1,4-di-N-oxide*. This compound was obtained in 6.5% yield from isopropyl benzofuroxane-5-carboxylate and ethyl 4,4,4-trifluoroacetoacetate. IR (KBr): 2981 (C-H), 1736 and 1708 (C=O), 1331 (*N*-oxide) cm^−1^. ^1^H-NMR (400 MHz, DMSO-*d*_6_) δ ppm: 1.39 (s, 9H, (CH_3_)_2_CH and CH_2_CH_3_), 4.5 (d, *J* = 6.30 Hz, 2H, CH_2_CH_3_), 5.21–5.25 (m, (CH_3_)_2_-CH-), 8.34 (d, *J* = 8.75 Hz, 1H, H5), 8.51 (d, *J* = 8.37 Hz, 1H, H6), 8.85 (s, 1H, H8). Calculated analysis for C_17_H_17_F_3_N_2_O_5_: C, 52.85; H, 4.44; N, 7.25. Found: C, 52.51; H, 4.05; N, 6.90.

**T-117**: *Isopropyl tert-butyl-3-heptafluoropropylquinoxaline-2,7-dicarboxylate 1,4-di-N-oxide*. This compound was obtained in 3.0% yield from isopropyl benzofuroxane-5-carboxylate and 6,6,7,7,8,8,8-heptafluoro-2,2-dimethyl-3,5-octanedione. IR (KBr): 2980 (C-H), 1733 and 1714 (C=O), 1332 (*N*-oxide) cm^−1^. ^1^H-NMR (400 MHz, DMSO-*d*_6_) δ ppm: 1.38 (s, 6H, (CH_3_)_2_CH), 1.42 (s, 9H, COC(CH_3_)_3_), 5.21–5.24 (m, 1H, (CH_3_)_2_CH), 8.38 (d, *J* = 9.01 Hz, 1H, H5), 8.52 (m, 1H, H6), 8.87 (s, 1H, H8). Calculated analysis for C_20_H_19_F_7_N_2_O_6_: C, 48.01; H, 3.83; N, 5.60. Found: C, 47.85; H, 3.56; N, 5.34.

**T-118**: *Isopropyl 2-pentafluorocarbonyl-3-trifluoromethylquinoxaline-7-carboxylate 1,4-di N-oxide*. This compound was obtained in 4.0% yield from isopropyl benzofuroxane-5-carboxylate and 1,1,1,5,5,6,6,6-octafluoro-2,4-hexanedione. IR (KBr): 2986 (C-H), 1728 (C=O), 1332 (*N*-oxide), 1284 and 1152 (CF_3_) cm^−1^. ^1^H-NMR (400 MHz, DMSO-*d*_6_) δ ppm: 1.38 (s, 6H, (CH_3_)_2_CH), 5.22–5.26 (m, (CH_3_)_2_-CH-), 8.51 (d, *J* = 9.5 Hz, 1H, H5), 8.62 (d, *J* = 9.4 Hz, 1H, H6), 8.9 (s, 1H, H8). Calculated analysis for C_16_H_10_F_8_N_2_O_5_: C, 41.57; H, 2.18; N, 6.06. Found: C, 41.16; H, 2.05; N, 5.84.

**T-124**: *n-Propyl 2-propionyl-3-trifluoromethylquinoxaline-7-carboxylate 1,4-di-N-oxide*. This compound was obtained in 7.52% yield from *n*-propyl benzofuroxane-5-carboxylate and 1,1,1-trifluoro-2,4-hexanedione. IR (KBr): 2981 (Ar C-H), 1724 (C=O), 1329 (*N*-oxide), 1256 (Ar-CF_3_) cm^−1^. ^1^H-NMR (400 MHz, DMSO-*d*_6_) δ ppm: 1.0 (t, 3H, CH_3_(CH_2_)_2_O), 1.35 (t, 3H, COOCH_2_CH_3_), 1.8 (q, *J* = 7.11, *J* = 14.15, 2H, CH_3_(CH_2_)_2_O), 4.3–4.35 (m, 2H, CH_3_(CH_2_)_2_O), 4.5 (q, *J* = 7.09 2H, COOCH_2_CH_3_), 8.40–8.45 (m, 1H, H5), 8.55 (d, *J* = 9.0 Hz, 1H, H6), 8.9 (s, 1H, H8).Calculated analysis for C_16_H_15_F_3_N_2_O_5_: C, 51.62; H, 4.06; N, 7.52. Found: C, 51.4; H, 3.85; N, 7.20.

**T-125**: *n-Propyl 2-phenylamide-3-methylquinoxaline-7-carboxylate 1,4-di-N-oxide*. This compound was obtained in 12.36% yield from *n*-propyl benzofuroxane-5-carboxylate and 3-oxo-*N*-phenylbutanamide. IR (KBr): 3245 (N-H), 2972 (Ar C-H), 1720 (C=O), 1328 (*N*-oxide) cm^−1^. ^1^H-NMR (400 MHz, DMSO-*d*_6_) δ ppm: 1.02 (t, 3H, CH_3_(CH_2_)_2_O), 1.8 (q, *J*_1_ = 6.97 Hz, *J*_2_ = 13.90 Hz, 2H, CH_3_(CH_2_)_2_O), 2.51 (s, 1H, CH_3_), 4.36 (t, *J* = 6.29 Hz, CH_3_(CH_2_)_2_O), 7.20 (t, *J* = 7.35 Hz, 2H, H3 and H5, NHC_6_H_5_), 7.42 (t, *J* = 7.47 Hz, 1H, H4, NHC_6_H_5_), 7.67 (d, *J* = 7.83 Hz, 2H, H2 and H6, NHC_6_H_5_)) 8.4 (d, *J* = 8.88 Hz, 1H, H5), 8.6 (d, *J* = 8.82 Hz, 1H, H6), 9.0 (s, 1H, H8), 11.02 (s, 1H, NH). Calculated analysis for C_20_H_19_N_3_O_5_: C, 62.99; H, 5.02; N, 11.02. Found: C, 62.60; H, 4.85; N, 10.70.

**T-126**: *n-Propyl 2-acetyl-3-methylquinoxaline-7-carboxylate 1,4-di-N-oxide*. This compound was obtained in 8.95% yield from *n*-propyl benzofuroxane-5-carboxylate and 2,4-pentanedione. IR (KBr): 2984 (Ar C-H), 1726 (C=O), 1330 (*N*-oxide), 1258 (Ar-CF_3_) cm^−1^. ^1^H-NMR (400 MHz, DMSO-*d*_6_) δ ppm: 1.0 (t, 3H, CH_3_(CH_2_)_2_O), 1.8 (q, *J*_1_ = 6.97 Hz, *J*_2_ = 13.90 Hz, 2H, CH_3_(CH_2_)_2_O), 2.55 (s, 1H, CH_3_), 3.97 (s, 3H, CH_3_), 4.36 (t, *J* = 6.29 Hz, CH_3_(CH_2_)_2_O), 8.40 (d, *J* = 8.17 Hz, 2H, H2 and H6, C_6_H_5_), 8.5 (d, *J* = 8.84, 2H, H5 and H6), 8.92 (s, 1H, H8). Calculated analysis for C_15_H_16_N_2_O_5_: C, 59.21; H, 5.30; N, 9.21. Found: C, 58.95; H, 5.15; N, 8.85.

**T-130**: *n-Propyl 2-amide-3-methylquinoxaline-7-carboxylate 1,4-di-N-oxide*. This compound was obtained in 5.68% yield from *n*-propyl benzofuroxane-5-carboxylate and acetoacetamide. IR (KBr): 2976 (Ar C-H), 1728 (C=O), 1334 (*N*-oxide), 1254 (Ar-CF_3_) cm^−1^. ^1^H-NMR (400 MHz, DMSO-*d*_6_) δ ppm: 0.99 (t, 3H, CH_3_(CH_2_)_2_O), 1.8 (q, *J*_1_ = 7.08 Hz, *J*_2_ = 14.10 Hz, 2H, CH_3_(CH_2_)_2_O), 2.47 (s, 3H, CH_3_), 4.4 (t, *J* = 6.58 Hz, CH_3_(CH_2_)_2_O), 8.27 (s, 2H, NH_2_), 8.39 (d, *J* = 8.97 Hz, 2H, H2 and H6, C_6_H_5_), 8.6 (d, *J* = 8.87, 2H, H5 and H6), 8.96 (s, 1H, H8). Calculated analysis for C_14_H_15_N_3_O_5_: C, 55.08; H, 4.95; N, 13.76. Found: C, 54.75; H, 4.62; N, 13.35.

### 3.2. Biological Assays

#### 3.2.1. Parasites

In this study, two strains of *T. cruzi* isolated in Mexico were used. *T. cruzi* NINOA strain (MHOM/MX/1994/NINOA) was obtained from a patient with acute Chagas disease [32] and *T. cruzi* INC-5 (MHOM/MX/1994/INC5) was isolated from a patient in the chronic phase of the disease [33]. Epimastigotes were cultured in vitro in liver infusion-tryptose (LIT) medium supplemented with 10% heat inactivated fetal bovine serum (FBS) (Gibco) and 100 U/mL penicillin-streptomycin (In vitro S.A.), and incubated at 27 °C. CD1 mice, aged 6–8 weeks, were infected with *T. cruzi* to obtain bloodstream trypomastigote samples. Animal experiments were performed according to Norma Oficial Mexicana (NOM-062-Z00-1999) published on 22 August, 2009.

#### 3.2.2. Evaluation on Epimastigotes and IC_50_

Viability assays of *T. cruzi* epimastigotes using 3-(4,5-dimethylthiazol-2-yl)-2,5-diphenyltetrazolium bromide (MTT) were performed. Briefly, the parasites were harvested during the exponential growth phase for a period of five days. In a 96-well microplate 1x10^6^ epimastigotes were deposited in brain-heart infusion (BHI) medium supplemented with 10% FBS and 100 U/mL antibiotic-antifungal mixture (Gibco). Five concentrations serial twofold from 50 μg/mL to 3.12 μg/mL for each of the quinoxaline 1,4-di-*N*-oxide derivatives and the reference drugs (Nfx and Bnz) were evaluated. Each well reached a final volume of 100 µL. Each condition was assessed in triplicate. The microplate was incubated for 24 h at 27 °C in darkness. Afterwards, 10 μL of a 5 mg/mL MTT solution was added and the plate was incubated for 20 h at 27 °C. Then 200 μL of 10% dodecyl sulfate (SDS)/0.1% HCl solution was added to dissolve formazan salts; the plate was incubated an additional 4 h. Finally, the plate was read at 570 nm in a spectrophotometer (Multiskan EX Thermo Electron, Taipei, Taiwan). The amount of viable cells was proportional to the amount of formazan produced. The inhibitory concentration of 50% of the population (IC_50_) was determined by the Probit statistical tool. Later the results were converted to micromolar units. Compounds with an IC_50_ less or equal to 10 µM were selected. As a positive control, parasites were incubated only in culture medium with a 1% DMSO concentration, corresponding to the highest sample dilution of DMSO [34,35,36].

#### 3.2.3. Evaluation on Bloodstream Trypomastigotes and LC_50_

CD1 female mice 6–8 weeks old were infected with *T. cruzi* bloodstream trypomastigotes of the INC-5 and NINOA strains. The course of infection continued for 4–6 weeks. At the peak of parasitemia blood was obtained by cardiac puncture using sodium heparin as an anticoagulant. The blood was adjusted to 1 × 10^6^ bloodstream trypomastigotes/mL. 

In each well of the 96-well plate, 195 μL of infected blood and 5 μL of quinoxaline 1,4-di-*N*-oxide derivatives or reference drug dilutions were seeded, with each well reaching a final volume of 200 µL. Each assay was performed in triplicate. Initially, all compounds were tested at 50 μg/mL. Afterwards, the compounds with a percentage of lysis >50 were tested at five concentrations (100 to 10 μg/mL) to obtain a lysis concentration of 50% of the population (LC_50_). As a negative control of lysis, wells with untreated blood trypomastigotes were used, and as a positive control, wells with the reference drugs were used. The microplates were incubated at 4 °C for 24 h. Bloodstream trypomastigotes were quantified by the method of Brener-Pizzi, 5 μL of blood were placed on a slide and covered with a coverslip 13 × 13 mm. The mobile protozoa were counted in 15 fields at 40× magnification using an optical microscope. The percentage lysis was determined by comparing the remaining trypomastigotes against the negative control. LC_50_ was determined with the Probit statistical tool. The results were later converted to micromolar units [23,37,38].

#### 3.2.4. Cytotoxicity Assay and CC_50_

The murine macrophage cell line J774.A1 was maintained in culture flasks with RPMI 1640 medium supplemented with 10% FBS, 1% MEM-NEAA medium (Gibco) and 100 U/mL antibiotic-antifungal mixture (Gibco). Cells were incubated at 37 °C with 5% CO_2_ and humidity. Trypsinized J774.A1 macrophages were washed and viability was assessed by the trypan blue dye (Sigma-Aldrich, Toluca, Edo de Mexico, Mexico) exclusion assay. In a 96-well microplate, 50,000 macrophages were seeded per well and a dose-response assay was performed. Compounds were evaluated using six-fold serial dilutions ranging from 100 µg/mL to 0.3 µg/mL of final concentration. Each compound was tested in triplicate. After 20 h incubation, Alamar Blue was added (10% *v*/*v*) and cells were incubated for an additional 4 h period. Results were obtained as previously described and CC_50_ was calculated. As a negative control of cytotoxicity, cells were incubated only in the presence of 0.1% DMSO [39,40].

#### 3.2.5. Determination of the SI and Selection of Active Compounds

The selectivity index (SI) was calculated to evaluate the selectivity of the molecules for the parasite. SI is defined as the ratio of CC_50_/IC_50_ [41].

### 3.3. Molecular Docking

*In silico* molecular docking studies were performed with Vina software [42] in order to predict the binding energy of the quinoxaline 1,4-di-*N*-oxide derivatives on the active site of the TR protein from *T. cruzi*. The TR crystal structure with PDBID: 1BZL [27] was downloaded from the protein databank (www.rcsb.org). This protein exists co-crystalized with its natural substrate trypanothione disulfide, which is located on the active site formed by the interface of the two dimers that comprise the whole protein. The crystal structure was prepared as a receptor by removing the waters and substrates and adding the Gasteiger charges using AutoDock software [43]. Then the location of the natural ligand on the active site was inspected to obtain the optimal grid box using the AutoDock graphical interface. The grid box size employed was 17 Å for each X, Y, and Z dimension and separated by 1 Å grid points; the center was located at 24.914, 10.253, −5.117 for X, Y, and Z dimensions, respectively. The grid box coordinates were used to dock the quinoxaline 1,4-di-*N*-oxide derivatives including trypanothionine. The compounds were drawn and energy minimized with MarvinSketch software (www.chemaxon.com). The hydrogens were added with babel software and the Gasteiger charges were added during the transformation to pdbqt format with the “prepare-ligand.py” script from AutoDock tools. For each docked ligand the predicted binding energy reported in Vina scores (the summation includes all of the pairs of atoms that are move relative to each other) [42] was used to rank the best TR protein inhibitors in comparison with the natural trypanothione substrate. Compounds with higher binding-energy values than the substrate TR are weak-binding ligands and those with lower binding-energy values are potential stronger-binding ligands. The molecular weight for each compound was also calculated with MarvinSketch software. The mean, standard deviations, Spearman´s correlation and the independent-sample *t-*test were performed with R software [44]. 

### 3.4. Enzyme Inhibitor Studies

Trypanothione disulfide (TS_2_) and recombinant *T. cruzi* TR were prepared following published procedures [45,46]. Recombinant human glutathione reductase (GR) was kindly provided by Dr. Heiner Schirmer, Heidelberg Germany. Stock solutions (5 mM) of the inhibitors were prepared in DMSO. The kinetic analyses were conducted using a Jasco V650 spectrophotometer. All enzymatic tests were performed in polystyrene optical cuvettes (10 × 4 × 45 mm). TR activity was measured at 25 °C in a total volume of 1 mL TR assay buffer (40 mM HEPES, 1 mM EDTA, pH 7.5 [47]) containing 100 μM NADPH and 5–10 mU enzyme in the absence and presence of the inhibitor. Each assay contained a total of 5% DMSO. The reaction was started by adding TS_2_ and NADPH consumption was followed at 340 nm.

GR activity was measured at 25 °C in a total volume of 1 mL GR assay buffer (20.5 mM KH_2_PO_4_, 26.5 mM K_2_HPO_4_, 200 mM KCl, 1 mM EDTA, pH 6.9). The assays contained 100 µM NADPH and 5–10 mU GR and varying concentrations of the inhibitor. The reaction was started by adding 100 µM GSSG and the absorption decrease was followed at 340 nm.

#### 3.4.1. IC_50_ Determination

Five compound concentrations were used ranging from 100–5 μM. All measurements were performed twice in three independent series. Enzyme activity was plotted versus increasing inhibitor concentrations. IC_50_ values were calculated using the four-parameter equation model 205 and the option “unlock” from the XLfit add-in (IDBS, Guildford, United Kingdom) for Excel (Microsoft Corporation, Redmond, WA, USA).

#### 3.4.2. Determination of the Type of Inhibition

The type of inhibition and the inhibitor constants were derived from Lineweaver-Burk plot. The activity of TR was measured in the absence and presence of two to three constant concentrations of inhibitor varying the concentration of TS_2_ (20, 40, 60, 100 and 200 µM). The inhibitory constant Ki was calculated from the direct plot using nonlinear least-squares data fitting in Excel spreadsheet [48,49].

## 4. Conclusions

In this study, in vitro evaluations of propyl and isopropyl 7 quinoxaline carboxylate 1,4-di-*N-*oxide derivatives against epimastigotes and trypomastigotes of *T. cruzi* were made. Four compounds were active against both forms of the parasite; one of them, **T-085**, showed a better trypanocidal activity than the reference drugs, exhibited a moderate selectivity against the epimastigote and bloodstream tripomastigotes life cycle of the parasite and a high affinity in docking and enzymatic analysis for TR. The latter demonstrated that **T-085** acts as a noncompetitive inhibitor. Structurally, the presence of an isopropyl carboxylate group in R7, a trifluoromethyl group in R3, and a carbonyl group in R2 of quinoxaline 1,4-di-*N-*oxide derivatives, favors its anti-trypanocidal activity as a TR inhibitor.

## Figures and Tables

**Table 1:**
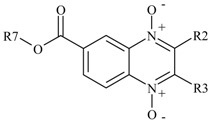
Structure and in vitro biological activities of quinoxaline 1,4-di-*N*-oxide on *T. cruzi* INC-5 epimastigotes and the J774A.1 macrophage cell line.

Compound	R2	R3	R7	IC_50_ (µM)	CC_50_ (µM)	SI
**Bnz**				42.34 ± 5.76	352.01 ± 17.1	8.31
**Nfx**				8.74 ± 3.8	201.05 ± 12.5	22.98
T-064	COOCH_3_	CH_3_	(CH_3_)_2_CH	43.66 ± 8.6	135.04 ± 11.1	3.09
T-065	COOCH_2_CH_3_	CH_3_	(CH_3_)_2_CH	>147.86	>295.73	Nd ^a^
T-066	COOC(CH_3_)_3_	CH_3_	(CH_3_)_2_CH	>136.55	>273.09	Nd ^a^
T-067	COOCH_2_CH_3_	CH_2_COOCH_2_CH_3_	(CH_3_)_2_CH	12.12 ± 0.4	44.37 ± 7.1	3.66
**T-069**	**COC_4_H_3_S**	**CF_3_**	**(CH_3_)_2_CH**	**4.95 ± 0.5**	12.80 ± 0.6	2.58
**T-070**	**COCH_3_**	**CF_3_**	**(CH_3_)_2_CH**	**2.83 ± 0.7**	17.50 ± 0.7	6.17
**T-071**	**COC_6_H_5_**	**CF_3_**	**(CH_3_)_2_CH**	**4.03 ± 0.5**	6.70 ± 0.8	1.66
T-072	COC_10_H_7_	CF_3_	(CH_3_)_2_CH	46.51 ± 7.5	15.14 ± 0.6	0.32
T-073	COC_4_H_3_O	CF_3_	(CH_3_)_2_CH	33.66 ± 9.2	55.35 ± 2.9	1.64
**T-085**	**COCH(CH_3_)_2_**	**CF_3_**	**(CH_3_)_2_CH**	**2.42 ± 0.5**	10.38 ± 0.8	4.28
T-088	COOCH_3_	CH_3_	CH_3_CH_2_CH_2_	138.55 ± 13.5	143.00 ± 9.7	1.03
**T-089**	**COC_4_H_3_S**	**CF_3_**	**CH_3_CH_2_CH_2_**	**7.59 ± 1.4**	7.71 ± 0.7	1.01
T-090	COOCH_2_CH_3_	CH_3_	CH_3_CH_2_CH_2_	155.14 ± 12.3	145.50 ± 9.2	0.93
T-091	COOC(CH_3_)_3_	CH_3_	CH_3_CH_2_CH_2_	>136.55	>273.09	Nd ^a^
T-097	CONHC_6_H_5_	CH_3_	(CH_3_)_2_CH	>130.50	>261.00	Nd ^a^
T-098	COC_6_H_5_	CH_3_	(CH_3_)_2_CH	141.90 ± 8.3	139.62 ± 8.1	0.98
T-107	COC(CH_3_)_3_	C(CH_3_)_3_	(CH_3_)_2_CH	91.71 ± 10.5	164.43 ± 7.7	1.79
T-108	CONH_2_	CH_3_	(CH_3_)_2_CH	130.67 ± 12.3	221.35 ± 9.4	1.69
**T-116**	**COOCH_2_CH_3_**	**CF_3_**	**(CH_3_)_2_CH**	**5.20 ± 2.1**	14.51 ± 0.8	2.78
T-117	COC(CH_3_)_3_	CF_2_CF_2_CF_3_	(CH_3_)_2_CH	100.03 ± 7.9	138.89 ± 5.6	1.38
T-118	COCF_2_CF_3_	CF_3_	(CH_3_)_2_CH	72.17 ± 8.0	157.39 ± 6.3	2.18
**T-124**	**COOCH_2_CH_3_**	**CF_3_**	**CH_3_CH_2_CH_2_**	**4.21 ± 0.5**	8.77 ± 3.4	2.08
T-125	CONHC_6_H_5_	CH_3_	CH_3_CH_2_CH_2_	>130.50	>261.00	Nd ^a^
T-126	COCH_3_	CH_3_	CH_3_CH_2_CH_2_	>163.33	>326.67	Nd ^a^
T-130	CONH_2_	CH_3_	CH_3_CH_2_CH_2_	238.28 ± 11.5	>325.61	Nd ^a^

The data represent the mean and SD of triplicate experiments. Nd ^a^: Not determined

**Table 2:** Biological activity of quinoxaline 1,4-di-*N*-oxide on *T. cruzi* trypomastigotes.

Compound	% Lysis at 50 μg/mL		LC_50_ (µM)	
	NINOA Strain	INC-5 Strain	NINOA Strain	INC-5 Strain
Bzn	58.10 ± 4.5	53.50 ± 5.6	98.83.72 ± 17.7	85.26 ± 16.7
Nfx	67.30 ± 5.4	62.60 ± 3.9	117.34 ± 17.7	113.86 + 17.8
T-064	3.20 ± 2.0	1.70 ± 2.0	Nd ^a^	Nd ^a^
T-065	0.77 ± 1.58	3.78 ± 5.7	Nd ^a^	Nd ^a^
T-066	2.60 ± 4.3	5.40 ± 3.7	Nd ^a^	Nd ^a^
T-067	18.50 ± 2.2	14.38 ± 9.2	Nd ^a^	Nd ^a^
T-069	57.33 ± 2.8	77.74 ± 9.3	98.03 ± 6.1	110.8 ± 9.3
T-070	45.97 ± 2.7	10.82 ± 5.4	Nd ^a^	Nd ^a^
T-071	58.79 ± 4.4	69.18 ± 5.1	97.8 ± 6.3	103.2 ± 8.3
T-072	17.77 ± 2.3	4.11 ± 3.8	Nd ^a^	Nd ^a^
T-073	35.05 ± 4.5	22.10 ± 4.32	Nd ^a^	Nd ^a^
**T-085**	**74.17 ± 6.4**	**76.20 ± 1.2**	**59.9 ± 7.9**	**73.1 ± 12.4**
T-088	16.26 ± 11.1	33.56 ± 6.8	Nd ^a^	Nd ^a^
T-089	51.65 ± 3.4	41.98 ± 6.5	114.7 ± 8.4	122.1 ± 6.5
T-090	28.57 ± 3.1	47.26 ± 3.3	Nd ^a^	Nd ^a^
T-091	7.30 ± 3.3	8.15 ± 2.2	Nd ^a^	Nd ^a^
T-097	12.18 ± 10.9	12.37 ± 5.2	Nd ^a^	Nd ^a^
T-098	6.89 ± 5.8	0.34 ± 0.6	Nd ^a^	Nd ^a^
T-107	6.28 ± 4.7	5.32 ± 3.8	Nd ^a^	Nd ^a^
T-108	12.80 ± 7.2	18.80 ± 2.8	Nd ^a^	Nd ^a^
T-116	49.88 ± 4.0	17.64 ± 2.6	Nd ^a^	Nd ^a^
T-117	40.51 ± 4.1	10.31 ± 4.9	Nd ^a^	Nd ^a^
T-118	34.39 ± 3.1	0.00 ± 3.2	Nd ^a^	Nd ^a^
T-124	24.61 ± 4.3	13.40 ± 8.9	Nd ^a^	Nd ^a^
T-125	35.62 ± 3.6	0.00 ± 1.4	Nd ^a^	Nd ^a^
T-126	17.69 ± 3.1	0.00 ± 6.1	Nd ^a^	Nd ^a^
T-130	4.65 ± 3.4	0.69 ± 1.2	Nd ^a^	Nd ^a^

The data represent the mean and SD of triplicate experiments. Nd ^a^: Not determinated

**Table 3:** Predicted binding affinities of the compounds docked on the active site of trypanothione reductase (TR) and their molecular weights (MW).

Compound	Vina Score	MW
T-072	−8.6	472.124
T-097	−7.7	383.148
T-098	−7.7	368.137
T-071	−7.6	422.108
T-073	−7.4	412.088
T-069	−7.3	410.168
T-003	−7.2	340.105
T-117	−7.2	502.133
T-118	−7.2	464.061
T-125	−7.2	383.148
T-070	−7.1	360.093
**T-085**	**−7.1**	**388.124**
T-089	−6.9	428.065
*Trypanothionine*	−*6.9*	*721.288*
T-107	−6.7	391.223
T-108	−6.7	307.116
T-116	−6.7	392.119
T-066	−6.6	366.179
T-021	−6.5	322.152
T-044	−6.5	279.085
T-130	−6.5	307.116
T-045	−6.4	293.101
T-126	−6.4	306.121
T-064	−6.2	324.132
T-067	−6.2	410.168
T-065	−6.1	338.147
T-090	−6.1	338.147
T-091	−6.1	366.179
T-124	−6.1	392.119
T-088	−5.9	324.132

**Table 4:** Inhibition of *T. cruzi* TR by quinoxaline-7-carboxylate 1,4-di-*N*-oxide derivatives.

Compound	Inhibitor [µM]	% Inhibition of TR at	
		100 µM [TS_2_]	40 µM [TS_2_]
T-085	5	15.2	20.3
	10	24.7	34.1
	20	36.1	45.7
	40	48.1	55.1
	60	60.1	60.9
	80 *	33	44
T-003	20	6	6.6
	40	10	9
	60	12	14.8
	80	18	20.5
	100	19.3	23.8
T-021	40	0	0
	100	0	7.5
T-044	40	0	0
	100	0	0

* precipitates in the assay.

## References

[1] Bern C. (2015). Chagas disease. N. Engl. J. Med..

[2] Shikanai-Yasuda M.A., Barbosa Carvalho N. (2012). Oral transmission of Chagas disease. Clin. Infec. Dis..

[3] Rassi A., Rassi A., Marin-Neto J.A., Franco-Paredes C., Santos-Preciado J.I. (2015). Chagas disease. Neglected Tropical Diseases-Latin America and the Caribbean.

[4] World Health Organization, Chagas Disease (American Trypanosomiasis). http://www.who.int/mediacentre/factsheets/fs340/en/.

[5] Nunes M.C., Dones W., Morillo C.A., Encina J.J., Ribeiro A.L. (2013). Chagas Disease: An overview of clinical and epidemiological aspects. J. Am. Coll. Cardiol..

[6] Belaunzarán M.L. (2015). Enfermedad de Chagas: Globalización y nuevas esperanzas para su cura. Rev. Argent. Microbiol..

[7] Sosa Estani S., Altcheh J., Riarte A., Freilij H., Fernandez M., Lloveras S., Pereiro A., Castellano L.G., Salvatella R., Nicholls R.S. (2015). Lineamientos básicos del tratamiento etiológico de la enfermedad de Chagas. Medicina (B. Aires).

[8] Brunton L., Lazo J., Parker K. (2012). Las Bases Farmacológicas de la Terapéutica: Goodman & Gilman.

[9] Murta S.M., Gazzinelli R.T., Brener Z., Romanha A.J. (1998). Molecular characterization of susceptible and naturally resistant strains of *Trypanosoma. cruzi* to benznidazole and nifurtimox. Mol. Biochem. Parasitol..

[10] Mejía-Jaramillo A.M., Fernández G.J., Montilla M., Nicholls R.S., Triana-Chávez O. (2012). Sensibilidad al benzonidazol de cepas de *Trypanosoma. cruzi* sugiere la circulación de cepas naturalmente resistentes en Colombia. Biomedica.

[11] Mejía A.M., Hall B.S., Taylor M.C., Gómez-Palacio A., Wilkinson S.R., Triana-Chávez O., Kelly J.M. (2012). Benznidazole-Resistance in *Trypanosoma. cruzi* is a readily acquired trait that can arise independently in a single Population. J. Infec. Dis..

[12] Guzmán-Marín E.S., Acosta-Viana K.Y., Jiménez-Coello M. (2016). La Enfermedad de Chagas: Retos del tratamiento. Biomédica.

[13] Vicente E., Pérez-Silanes S., Lima L.M., Ancizu S., Burguete A., Solano B., Villar R., Aldana I., Monge A. (2009). Selective activity against *Mycobacterium. tuberculosis* of new quinoxaline 1,4-di-*N*-oxides. Bioorg. Med. Chem..

[14] Duque-Montano B.E., Gomez-Caro L.C., Sanchez-Sanchez M., Monge A., Hernandez-Baltazar E., Rivera G., Torres-Angeles O. (2013). Synthesis and in vitro evaluation of new ethyl and methyl quinoxaline-7-carboxylate 1,4-di-*N-*oxide against *Entamoeba histolytica*. Bioorg. Med. Chem..

[15] Carta A., Loriga M., Paglietti G., Mattana A., Fiori P.L., Mollicotti P., Sechi L., Zanetti S. (2004). Synthesis, anti-mycobacterial, anti-trichomonas and anti-candida in vitro activities of 2-substituted-6,7-difluoro-3-methyl quinoxaline 1,4-dioxides. Eur. J. Med. Chem..

[16] Marin A., Moreira-Lima L., Solano B., Vicente E., Perez-Silanes S., Maurel S., Michel S., Aldana I., Monge A., Deharo E. (2008). Antiplasmodial structure-activity relationship of 3-trifluoromethyl-2-arylcarbonyl quinoxaline1,4-di-*N*-oxide derivatives. Exp. Parasitol..

[17] Estevez Y., Quiliano M., Burguete A., Cabanillas B., Zimic M., Malaga E., Verástegui M., Pére-Silanes S., Aldana I., Castillo D. (2011). Trypanocidal properties, structure-activity relationship and computational studies of quinoxaline 1,4-di-*N*-oxide derivatives. Exp. Parasitol..

[18] Cerecetto H., di Maio R., González M., Risso M., Saenz P., Seoane G., Denicola A., Peluffo G., Quijano C., Olea-Azar C. (1999). 1,2,5-Oxadiazole *N*-oxide derivatives and related compounds as potential antitrypanosomal drugs: structure-activity relationships. J. Med. Chem..

[19] Aguirre G., Cerecetto H., di Maio R., González M., Alfaro M.E., Jaso A., Zarranz B., Ortega M.A., Aldana I., Monge-Vega A. (2004). Quinoxaline *N*,*N*′-dioxide derivatives and related compounds as growth inhibitors of *Trypanosoma cruzi,* structure-activity relationships. Bioorg. Med. Chem..

[20] Ancizu S., Moreno E., Torres E., Burguete A., Pérez-Silanes S., Benítez D., Villar R., Solano B., Marín A., Aldana I. (2009). Heterocyclic-2-carboxylic acid (3-cyano-1,4-di-*N-*oxidequinoxalin-2-Yl)amide derivatives as hits for the development of neglected disease drugs. Molecules.

[21] Benitez D., Cabrera M., Hernández P., Boiani L., Lavaggi M.L., di Maio R., Yaluff G., Serna E., Torres S., Ferreira M.E. (2011). 3-Trifluoromethylquinoxaline *N,N’*-dioxides as anti-trypanosomatid agents. Identification of optimal anti-*T. cruzi* agents and mechanism of action studies. J. Med. Chem..

[22] Torres E., Moreno-Viguri E., Galiano S., Devarapally G., Crawford P.W., Azqueta A., Arbillaga L., Varela J., Birriel E., di Mairo R. (2013). Novel Quinoxaline 1,4-di-*N*-oxide Derivatives as new potential antichagasic agents. Eur. J. Med. Chem..

[23] Villalobos-Rocha J.C., Sánchez-Torres L., Nogueda-Torres B., Segura-Cabrera A., García-Pérez C.A., Bocanegra-García V., Palos I., Monge A., Rivera G. (2014). Anti-*Trypanosoma cruzi* and anti-leishmanial activity by quinoxaline-7-carboxylate 1,4-di-*N*-oxide derivatives. Parasitol. Res..

[24] Lopez S.E., Romero A. (2011). Grupo trifluorometilo: Un sustituyente importante en química medicinal. Rev. Fac. Farm. UCV.

[25] Vega-Gomez M.C., Rolón M.S., Yaluff G., Cerecetto-Meyer H., González M. (2012). Modelos de evaluación biológica in vitro e in vivo utilizados en la búsqueda de fármacos antichagásicos. Enfermedad de Chagas: Estrategias en la búsqueda de nuevos medicamentos, una visión iberoamericana.

[26] Dos-Santos V.A.F.F., Leite K.M., da Costa-Siqueira M., Regasini L.O., Martinez I., Nogueira C.T., Kolos Galuppo M., Stolf B.S., Soares-Pereira A.M., Cicarelli R.M.B. (2013). Antiprotozoal Activity of Quinonemethide Triterpenes from *Maytenus ilicifolia* (*Celastraceae*.). Molecules.

[27] Bond C.S., Zhang Y., Berriman M., Cunningham M.L., Fairlamb A.H., Hunter W.N. (1999). Crystal structure of *Trypanosoma. cruzi* trypanothione reductase in complex with trypanothione, and the structure-based discovery of new natural product inhibitors. Structure.

[28] Laskowki R.A., Swindells M.B. (2011). LigPlot+: Multiple ligand-protein interaction diagrams for drug discovery. J. Chem. Inf. Model..

[29] Persch E., Bryson S., Todoroff N.K., Eberle C., Thelemann J., Dirdjaja N., Kaiser M., Weber M., Derbani H., Brun R. (2014). Binding to large enzyme pockets: small-molecule inhibitors of trypanothione reductase. Chem. Med. Chem..

[30] Gómez Caro L.C., Sánchez Sánchez M., Bocanegra García V., Monge A., Rivera G. (2011). Synthesis of quinoxaline 1,4-di-*N*-oxide derivatives on solid support using room temperature and microwave-assisted solvent-free procedures. Quim Nova.

[31] Galvao J., Davis B., Tilley M., Normando E., Duchen M.R., Cordeiro M.F. (2014). Unexpected low-dose toxicity of the universal solvent DMSO. FASEB J..

[32] Bosseno M.F., Barnabé C., Gastelum E.M., Kasten F.L., Ramsey J., Espinoza B., Breniere F. (2002). Predominance of Trypanosoma cruzi Lineage I in Mexico. J. Clin. Microbiol..

[33] Ruíz-Sánchez R., León M.P., Matta V., Reyes P.A., López R., Jay D., Monteón V.M. (2005). *Trypanosoma cruzi* isolates from Mexican and Guatemalan acute and chronic chagasic cardiopathy patients belong to *Trypanosoma cruzi* I. Mem. Inst. Oswaldo Cruz.

[34] Da Silva M.T., Silva-Jardim I., Portapilla G.B., de Lima G.M., Costa F.C., Anibal F.F., Thieman O.H. (2016). In vivo and in vitro auranofin activity against *Trypanosoma cruzi:* Possible new uses for an old drug. Exp. Parasitol..

[35] Cotinguiba F., Regasini L.O., da Silva Bolzani V., Debonsi H.M., Duó Passerini G., Barretto Cicarelli R.M., Kato M.J., Furlan M. (2009). Piperamides and their derivatives as potential anti-trypanosomal agents. Med. Chem. Res..

[36] Muelas-Serrano S., Nogal-Ruiz J.J., Gómez-Barrio A. (2000). Setting of a colorimetric method to determine the viability of *Trypanosoma cruzi*. Parasitol. Res..

[37] Brener Z. (1962). Therapeutic activity and criterion of cure on mice experimentally infected with *Trypanosoma cruzi*. Rev. Inst. Med. Trop Sao Paulo.

[38] Díaz-Chiguer D.L., Márquez-Navarro A., Nogueda-Torres B., León-Ávila G.L., Pérez-Villanueva J., Hernández-Campos A., Castillo R., Ambrosio J.R., Nieto-Meneses R., Yépez-Mulia L. (2012). In vitro and in vivo trypanocidal activity of some benzimidazole derivatives against two strains of *Trypanosoma cruzi*. Acta Trop.

[39] O’Brian J., Wilson I., Orton T., Pognan F. (2000). Investigation of the alamar blue (resazurin) fluorescent dye for the assessment of mammalian cell cytotoxicity. Eur. J. Biochem..

[40] Mahmoudvand H., Tavakoli R., Sharififar F., Minaie K., Ezatpour B., Jahanbakhsh S., Sharifi I. (2015). Leishmanicidal and cytotoxic activities of *Nigella sativa* and its active principle, thymoquinone. Pharm. Biol..

[41] Nunes Dos Santos R.A., Batista J., Rosa S.I., Torquato H.F., Bassi C.L., Ribeiro T.A., de Sousa P.T., Beserra A.M., Fontes C.J., da Silva L.E. (2011). Leishmanicidal effect of *Spiranthera odoratíssima* (Rutaceae) and its isolated alkaloid skimmianine occurs by a nitric oxide dependent mechanism. Parasitology.

[42] Trott O., Olson A.J. (2010). AutoDock Vina: improving the speed and accuracy of docking with a new scoring function, efficient optimization, and multithreading. J. Comput. Chem..

[43] Morris G.M., Ruth H., Lindstrom W., Sanner M.F., Belew R.K., Goodsell D.S., Olson A.J. (2009). AutoDock4 and AutoDockTools4: automated docking with selective receptor flexibility. J. Comp. Chem..

[44] R Development Core Team 2016 R: A Language and Environment for Statistical Computing. https://www.R-project.org..

[45] Sullivan F.X., Walsh C.T. (1999). Cloning, sequencing, overproduction and purification of trypanothione reductase from *T. cruzi*. Mol. Biochem. Parasit..

[46] Comini M.A., Dirdjaja N., Kaschel M., Krauth-Siegel R.L. (2009). Preparative enzymatic synthesis of trypanothione and trypanothione analogues. Int. J. Parasitol..

[47] Jockers-Scherübl M.C., Schirmer R.H., Krauth-Siegel R.L. (1989). Trypanothione reductase from *Trypanosoma. cruzi*. Catalytic properties of the enzyme and inhibition studies with trypanocidal compounds. Eur. J. Biochem..

[48] Kemmer G., Keller S. (2010). Nonlinear least-squares data fitting in Excel spreadsheets. Nat. Protoc..

[49] Brown A.M. (2001). A step-by-step guide to non-linear regression analysis of experimental data using a Microsoft Excel spreadsheet. Comput. Meth. Prog. Bio..

